# The association between nutrient intake, nutritional status and physical function of community-dwelling ethnically diverse older adults

**DOI:** 10.1186/s40795-020-00363-6

**Published:** 2020-08-25

**Authors:** Evans A. Asamane, Carolyn A. Greig, Janice L. Thompson

**Affiliations:** 1 School of Sports, Exercise and Rehabilitation Sciences, College of Life and Environmental Sciences, University of Birmingham, Birmingham, UK; 2 School Primary, Community and Social Care, Keele University, Keele, UK; 3 MRC-Arthritis Research UK Centre for Musculoskeletal Ageing Research, University of Birmingham, Birmingham, UK

**Keywords:** Nutrient intake, Physical function, Nutritional status, Ageing, Ethnic minority, Older adults, United Kingdom

## Abstract

**Background:**

There are limited longitudinal data regarding nutrient intake, nutritional status and physical function in community-dwelling ethnically diverse older adults. This study explored these variables and their relationship at baseline (*n* = 100) and 8-months’ follow-up (*n* = 81) among community-dwelling ethnically diverse older adults (≥60 years) in Birmingham, United Kingdom.

**Methods:**

Multiple-pass 24-h dietary recalls and the Mini Nutritional Assessment-Short Form assessed nutritional intake and status, respectively. Short Physical Performance Battery (SPPB) and handgrip strength measured physical function. Linear and multinomial regressions were used to predict relationships between physical function, nutritional status and nutrient intake.

**Results:**

Complete data were collected at baseline (*n* = 100) and 8-months’ follow-up (*n* = 81). Mean (SD) age was 70 (8.1) years (60% male), with 62% being obese. Statistically significant decreases in intakes of vitamin B6, vitamin B1, iron, folate, and magnesium occurred over time. Daily intake of all micronutrients except vitamin B12, phosphorus and manganese were below the Recommended Nutrient Intakes (RNI). SPPB (Z = -4.01, *p* < 0.001) and nutritional status (Z = -2.37, *p* = 0.018) declined over time. Higher SPPB scores at baseline (OR = 0.54 95% CI 0.35, 0.81) were associated with a slower decline in nutritional status.

**Conclusion:**

The observed declines and inadequate nutrient intakes in the absence of weight loss in just 8 months may pose serious challenges to healthy ageing, identifying an urgent need to re-evaluate and tailor appropriate dietary advice for this population. Additionally, the associations of nutrition and physical function observed in this study serves as an essential resource to design and implement community/faith-based interventions targeting early screening of nutritional status and physical function to ensure most older adults are assessed and treated accordingly.

## Background

The world population is increasing and ageing rapidly, with developed countries having the highest proportion of ageing populations compared to developing countries [1]. In the United Kingdom (UK), there was a total of 11.2 million people aged 65+ years, representing 18% of the population in 2016 [2]. It is projected that by 2030, one in five people will be aged 65+ years, comprising 21.8% of the population [3]. Furthermore, the UK population is becoming increasingly ethnically diverse, with ethnic minorities significantly ageing alongside the white population [4]. According to the findings from the 2011 Office for National Statistics (ONS), it is projected that by 2051, there will be 3.8 million Black Minority Ethnic (BME) people aged 65+ years and 2.8 million BME people aged 70+ years [4]. The ageing and increasing ethnic diversity of the UK population has implications not only to the quality of life of the individual but significantly impacts on health and social care systems, the economy and the lives of family members [2, 5, 6].

Furthermore, many BMEs are often faced with lower socio-economic status and disproportionate health inequalities, which accounts for the differences in health outcomes among ethnic minorities and the white European population [7–9]. For instance, as evident in the findings of the Health Survey England (HSE), the prevalence of obesity among Black African women is 38% (risk ratio 2.0), and 32.1% among Black Caribbean women (risk ratio 1.43) compared to 23% in the general white British population of women [10]. While recognising these differences in health outcomes, dietary habits of BMEs have been shown to become unhealthy mainly due to financial constraints, language barriers, age, availability of traditional foods, and years spent in the host country [11–13]. A study in the UK found that older BME women with a mean age of 70.5 years had inadequate intakes of vitamin D, selenium, magnesium and potassium, and attributed reasons such as age, reduced physical activity and dietary restrictions to be influencing dietary intake [11]. Considering the ultimate role of diet as a modifiable factor in obesity and related chronic diseases, and the limited literature regarding it, it is essential to study the trends in nutrient intake particularly in this population with a relatively high prevalence of obesity and diet-related chronic diseases [10]. Most studies exploring nutrient intakes in ethnic minorities have focused only on south Asian population, single ethnic groups, or mainly one sex limiting a clearer understanding of the differences shared within and between ethnic minorities [11, 14, 15]. The present study is an initial step towards increasing knowledge on the heterogeneity and gender differences in nutrient intakes over time among ethnic minority older adults living in super-diverse communities.

Besides nutrition, the promotion and maintenance of good physical function are crucial in improving quality of life, nutrient intake and reducing institutionalisation, hospitalisation and mortality among older adults [16–18]. Ethnic minority groups and older adults living in lower socio-economic environments often have a lower physical function as compared to the general population [19, 20]. Despite this, few studies have explored the risk factors, specifically the impact of nutrient intake and nutritional status on the maintenance of physical function in this population. Published findings from non-ethnic minority community-dwelling older adults have shown that good nutrition can improve and maintain higher physical function such as keeping up with activities of daily living and preventing falls [21, 22]. For instance, a study of 689 community-dwelling older adults with a mean age of 75.7 years, found that an increased dietary diversity was closely associated with better scores of activities of daily living, lower depression scores and increased quality of life [21]. Considering these and the relatively higher risk of physical function limitations within this population, it has become more pertinent to explore the profile of nutrition and physical function, and their association over time among community-dwelling, ethnically diverse older adults.

The few studies that have examined these variables within this population are usually cross-sectional, with many employing a subjective measure of physical function and providing limited information on dietary intake and nutritional status. Additionally, the cross-sectional nature of these studies also hinders a deeper understanding of the impact of the time element on the relationships between diet, nutritional status, and objectively measured physical function. The present study aimed to: 1) provide a clearer understanding of the profile of nutrient intake, nutritional status and objectively measured physical function over time; and 2) explore how nutrient intake and nutritional status are associated with physical function over time in this population.

## Methods

### Study design and setting

An observational longitudinal quantitative approach was used to examine nutrition and physical function over 8 months. The study was conducted in the Birmingham area, West Midlands, UK. In this geographical location, ethnic minorities (non-white) comprise 46.9% of the population, with the largest group being South Asians (comprising 22.5% of the total population), and it is projected that ethnic minorities will become the majority of the population by 2021 [23, 24].

### Participants and recruitment

The sample included community-dwelling, ethnically diverse older adults aged 60 years and older, and self-identifying as Pakistani, Indian, Bangladeshi, Caribbean and/or African. In addition to these, other inclusion criteria encompassed residing in the Birmingham area, non-institutionalised and with no known diagnosis of dementia or any mental illness that will impair participation. A convenience sample of 100 participants were recruited through community centres, age-well societies, faith/religious centres and other informal social events by word of mouth. The rationale was to recruit a more representative sample, thus maximum variation technique was introduced after recruiting 60 participants [25].

### Measures

There were four different contact points for data collection, two completed within 14 days at baseline and repeated in the same format after 8 months of follow-up. Socio-demographic data including date of birth, sex, marital status, educational status, faith, ethnicity, and years residing in the UK were collected using a bespoke questionnaire, at baseline and at follow-up (See Additional file 1). The Index of Multiple Deprivation (IMD) was computed as an indicator of deprivation using the participant’s postcode [26]. Weight was measured to the nearest 0.1 kg and height to the nearest 0.1 cm, using a Seca 899 digital scale and Seca 213 portable stadiometer, respectively. Body Mass Index (BMI) was calculated using the standard equation; weight (kg)/height^2^ (m^2^). Waist circumference (WC) was measured and recorded to the nearest 0.01 cm using a flexible retractable non-elastic tape.

#### Dietary assessment

Dietary intake was assessed using the multiple-pass 24-h recall, following standard procedures [27]. EAA, a trained nutritionist with relevant expertise in conducting dietary assessments, conducted all assessments with regular data checks from JLT, a Professor of Public Health Nutrition and Exercise, with extensive experience in dietary assessments in diverse populations. A culturally-appropriate food portion booklet containing ethnic minority foods, accompanied by standard household food measures, were used to estimate portion sizes [28]. In total, four 24-h recalls were collected, two non-consecutive days each at baseline and follow-up. In instances where participants had fasted within the previous 24 h, the record was discarded, and a new day assigned to retake the record (*n* = 17). All 24-h recall interviews were digitally recorded to enhance the quality of the data during data entry. Detailed recipes were gathered for home-cooked meals, and brand names and cooking methods were recorded during interviews. In addition, nutrient supplement usage was collected during dietary assessments. After each visit, the multiple-pass 24-h recall data were checked for accuracy using the interviews before entering these data for processing into a dietary software analysis package, Dietplan 7.0 (Forestfield Software Ltd., Horsham, West Sussex, UK). Dietplan 7 was appropriate as it utilises UK-specific databases such as McCance and Widdowson, as well as relevant ethnic minority food databases. In instances that a food item could not be found in the software databases, these were added to the database using food manufacturing websites and other food composition tables. Nutrient data obtained from the analysis were compared with the age and sex-specific UK Dietary Reference Values (DRV) [29].

#### Nutritional status

Nutritional status was assessed using the Mini-Nutritional Assessment-Short Form (MNA-SF). This is a simple to use, sensitive, reliable, and valid tool for rapid assessment of nutritional status of older adults [30]. Unlike the full MNA, the short form is more rapid (can be completed within 10 min) and equally reliable to the full MNA [31, 32]. Given the validity and quick administration, it was the preferred option to assess nutritional status as it reduces participant burden during data collection. Additionally, as this study was part of a larger study, MNA was the tool chosen to measure nutritional status [33]. The MNA-SF is composed of six major items and has a scoring of 0–14 points, with 0–7 classified as malnourished, 8–11 classified as at risk of malnutrition, and 12–14 classified as normal [32].

#### Physical function

The Short Physical Performance Battery (SPPB) was used to measure participants’ lower body extremity function [17, 34]. SPPB has been widely validated to measure accurate physical function and serves as a prediction of mobility, disability and mortality in older adults [16]. SPPB includes a balance test conducted in side by side, semi-tandem, and tandem positions, as well as 4-m walk test at one’s self-selected ‘natural’ pace, and a test of their ability to rise five times from a chair as quickly as possible. The maximum score from these three components makes up a total score of 12 on the SPPB; a score of 0 indicates the worst performance, while a score of 12 indicates the best performance.

Handgrip Strength (HGS) was also used to measure physical function. HGS was measured in kilogram (Kg) using a Jamar hand dynamometer (Sammons Preston, Inc., Bolingbrook, IL) per the NHANES protocol for measuring HGS in the standing position, with arms by the side [35]. Handle width was adjusted to hand size, and each measurement performed three times [36]. The standing position was chosen as it has been shown to produce maximal grip strength as compared to the other positions in measuring HGS [37].

### Statistical analysis

Nutrient density was computed by dividing the nutrient intake by the total energy intake and multiplying it by 1000 kcal/day [38]. Independent T-test and chi-square test were conducted to test for differences in continuous and categorical variables, respectively, at both time points. The Wilcoxon signed-rank test was used to test for median differences in instances where the continuous variables failed to meet the assumptions for a t-test. Prior to conducting the above analyses, normality was tested using the Shapiro-Wilk test and Q-Q plots. Marital status was later regrouped as married and not married, educational status was regrouped as educated and not educated, the number of diseases grouped as number of diseases ≤2 and number of diseases > 2, and ethnicities regrouped as South Asian (Pakistanis, Indians and Bangladeshis), African-Caribbean (Africans and Caribbean), and ‘other ethnicity,’ (mixed ethnicities). Given the smaller numbers in each group, these regroupings were done to satisfy the assumptions needed to run some of the analyses.

For energy and macronutrient intakes, both mean (SD) and median (IQR) were presented. Micronutrient intakes and all other variables that satisfied normally distributed characteristics were presented as mean (SD), while those that were not normally distributed were presented as median (IQR). Categorical variables were presented as number (N) and percentages. The percentage of nutrient intake of the RNI defined for that nutrient was computed to ascertain whether the participant met the recommendations [39]. One-way ANOVA and Kruskal Wallis were performed to examine any differences in nutrient intake by ethnicities and other socio-demographic characteristics.

A pairwise correlation was conducted, and the variables correlated with physical function (*p* < 0.05), were built into a hierarchical model to test the predictors of physical function (SPPB and HGS) while controlling for variables of interest. For longitudinal analysis regarding physical function, the difference in physical function between both time points was computed and classified into the following groups: improved, declined and stable. In logistic regression, the improved/stable and declined groups were used as outcome variables to test factors predicting changes in physical function at follow-up. Regarding nutritional status, the at-risk of malnutrition and the malnourished groups were combined to form malnourished/at-risk of malnutrition group, while the normal nutritional status group was maintained as originally defined. Changes in nutritional status were 1) those who remained in the malnourished/at-risk of malnutrition; 2) those who declined from the normal to the malnourished/at-risk of malnutrition group; 3) those who improved from malnourished/at-risk of malnutrition to the normal group; and 4) those who remained in the normal group. Multinomial regression was used to predict the membership of these four nutritional status groups. A sensitivity analysis was conducted to ascertain differences in nutrient intakes with or without supplement intakes at both time points. All statistical analyses were conducted using IBM SPSS version 25 (IBM Corp, Armonk, NY, 2012) with significance set at *p* < 0.05.

## Results

Socio-demographic and other health-related data for participants at baseline and after 8 months of follow-up are presented in Table 1. Of the 19 of participants who did not complete the study, 5 did not continue due to sickness, 8 were no longer interested, and the remaining 6 could not be reached during follow-up. At baseline, the mean (SD) age was 70.8 (8.1), with 59% male. Most of the participants were married and had a University or college degree.

**Table 1:** Socio-demographic characteristics and other health-related information at baseline and 8-months’ follow-up

Variables		Baseline (***n*** = 100)	Follow-up (***n*** = 81)
Age, years Mean (SD)		70.8 (8.1)	70.7 (8.2)
Male N (%)		59 (59.0)	50 (62.0)
Ethnicity N (%)	Pakistani	23 (23.0)	15 (18.5)
	Indian	7 (7.0)	5 (6.2)
	Bangladeshi	4 (4.0)	2 (2.5)
	Caribbean	41 (41.0)	38 (46.9)
	African	19 (19.0)	15 (18.5)
	Others	6 (6.0)	6 (7.4)
Marital status N (%)	Single	4 (4.0)	3 (3.7)
	Married	66 (66.0)	55 (67.9)
	Divorced	14 (14.0)	12 (14.8)
	Widowed	16 (16.0)	11 (13.6)
Faith/Religion N (%)	No religion	1 (1.0)	1 (1.2)
	Hindu	2 (2.0)	2 (2.5)
	Sikh	7 (7.0)	4 (5.0)
	Muslim	34 (34.0)	24 (29.6)
	Christian	56 (56.0)	50 (61.7)
Education N (%)	No education	16 (16.0)	11 (13.6)
	Primary	16 (16.0)	14 (17.3)
	Secondary	21 (21.0)	15 (18.5)
	College/University	47 (47.0)	41 (50.6)
Self-rated health N (%)	Excellent	18 (18.0)	16 (19.7)
	Good	55 (55.0)	45 (55.6)
	Fair	16 (16.0)	11 (13.6)
	Poor	11 (11.0)	9 (11.1)
No. of diseases Mean (SD)		2.0 (1.4)	2.1 (1.4)
IMD Decile N (%)	1 (Most deprived)	33 (33.0)	29 (35.8)
	2	19 (19.0)	19 (23.4)
	3	22 (22.0)	11 (13.6)
	4 (least deprived)	26 (26.0)	22 (27.2)
BMI categories* N (%)	Normal	7 (7.0)	9 (11.1)
	Overweight	31 (31.0)	21 (25.9)
	Obese	62 (62.0)	51 (63.0)
^**a**^Nutritional status by MNA-SF N(%)**	Malnourished	2 (2.0)	1 (1.2)
	At- risk of malnutrition	22 (22.0)	29 (35.8)
	Normal	76 (76.0)	51 (63.0)
^**a**^SPPB score Median (IQR) **		11.1 (4.0)	10.0 (4.0)
HGS Mean (SD)		27.6 (9.8)	26.5 (9.5)
WC Mean (SD)		100.3 (10.6)	100.8 (10.6)

*SD* Standard deviation, *IQR* Interquartile range, *IMD* Index of Multiple Deprivation, *BMI* Body Mass Index, *MNA-SF* Mini-Nutritional Assessment-Short Form, *HGS* Handgrip Strength, *WC* Waist circumference *WHO guidance on BMI thresholds for Asian populations (World Health Organization, 2004) was used to categorise BMI of South Asian participants, and the standard BMI categories were used for Caribbean and African participants. ** *p* < 0.05; ^a^ Wilcoxon Rank test used to examine median differences

Older adults completing the study had a significantly higher SPPB (*P* = 0.007), HGS (*p* = 0.027) and lower WC (*p* = 0.036) as compared to those lost to follow-up (*n* = 19). All other variables of interest were not significantly different (See Additional file 2).

### Energy and macronutrient intakes over time compared with DRVs

Mean (SD) and median (IQR) values for energy and nutrient intakes are reported in Table 2. Energy intakes for both sexes at both time points were below the estimated average requirement based on the population with low physical activity levels. There was a decrease in polyunsaturated fatty acids intake for females at follow-up (*P* = 0.04) (Table 2). The mean percentage Total Energy (%TE) from carbohydrates at baseline and follow-up for both sexes were slightly above the DRV. However, the %TE from fat at both time points for females were below the DRV (31.3 and 32.3%, respectively, vs 35%).

**Table 2:** Macronutrient and energy intake of community-dwelling, ethnically diverse older adults at baseline and follow-up (*N* = 81)

Nutrient intake	Baseline			Follow-up			***P***-values*
	Sex	Mean (SD)	Median (IQR)	Mean (SD)	Median (IQR)	RNI ^**a**^	
Energy Kcal/day	Male	1884.6 (518.2)	1853.8 (609.6)	1965.0 (686.5)	1822 (797.8)	2103.3–2366.2	0.393
	Female	1514.5 (428.3)	1358.5 (526.5)	1354.5 (498.5)	1243.5 (676.0)	1673.0–1888.1	0.065
Carbs g	Male	230.2 (61.9)	223.8 (60.9)	248.9 (72.1)	234.8 (83.0)	–	0.104
	Female	195.4 (60.9)	201.1 (95.3)	178.2 (74.0)	161.8 (84.5)		0.189
%TE Carbs	Male	50.2 (11.6)	48.2 (17.7)	53.5 (10.8)	52.7 (16.4)	50	0.101
	Female	51.8 (8.9)	50.2 (9.2)	52.1 (9.2)	52.3 (14.5)		0.883
Fibre g/day	Male	18.3 (7.3)	17.2 (10.0)	20.3 (10.8)	19.1 (10.5)	30	0.192
	Female	17.1 (7.9)	15.0 (10.7)	14.1 (7.5)	12.8 (9.5)		0.084
Protein g	Male	79.1 (25.3)	74.2 (28.7)	77.6 (34.4)	73.9 (41.5)		0.736
	Female	68.0 (23.0)	57.5 (36.6)	58.6 (23.4)	53.2 (28.2)		0.070
Protein g/kg/day	Male	1.02 (0.40)	0.94 (0.44)	0.98 (0.48)	0.90 (0.51)	1.0–1.2 g/kg [40]	0.754
	Female	0.93 (0.33)	0.86 (0.50)	0.80 (0.37)	0.76 (0.38)		0.183
%TE Protein	Male	17.0 (3.9)	16.8 (5.3)	15.8 (4.2)	15.3 (3.9)	15	0.127
	Female	18.1 (4.4)	17.7 (4.8)	18.0 (5.9)	18.0 (6.7)		0.912
Fats g	Male	75.3 (40.8)	73.6 (49.2)	73.5 (44.7)	66.6 (39.5)	–	0.816
	Female	53.3 (20.2)	53.0 (33.7)	48.8 (22.4)	41.6 (29.9)		0.319
%TE Fats	Male	34.3 (10.8)	36.3 (16.5)	38.1 (37.6)	32.4 (12.8)	35	0.453
	Female	31.6 (8.6)	31.3 (14.4)	32.3 (7.0)	31.1 (10.3)		0.724
Saturated fats g	Male	24.2 (14.5)	22.2 (17.5)	28.9 (28.0)	23.6 (17.2)	–	0.203
	Female	17.5 (8.9)	16.9 (12.4)	17.2 (10.7)	14.0 (8.5)		0.885
%TE Saturated fats	Male	11.2 (4.8)	11.0 (6.7)	11.3 (4.3)	11.1 (6.0)	11	0.885
	Female	10.4 (4.6)	9.7 (4.8)	11.2 (4.2)	10.8 (6.7)		0.445
MUFA g	Male	24 (14.0)	22.8 (17.2)	24.8 (16.9)	20.6 (15.2)	–	0.742
	Female	17.6 (8.3)	17.9 (12.1)	16.1 (9.2)	14.6 (8.8)		0.385
%TE MUFA	Male	11.1 (4.6)	11.3 (6.1)	10.3 (3.8)	10.6 (6.0)	12	0.310
	Female	10.4 (3.9)	10.4 (4.8)	10.5 (36)	10.4 (3.6)		0.889
PUFA g	Male	13.9 (10.2)	10.5 (8.8)	15.4 (14.6)	10.9 (13.5)		0.460
	Female	8.9 (4.1)	9.7 (7.1)	7.2 (3.7)	6.2 (3.6)		0.040
% TE PUFA	Male	6.3 (3.4)	5.7 (3.2)	6.7 (5.9)	5.5 (5.1)		0.626
	Female	5.2 (2.0)	4.9 (3.0)	4.8 (1.7)	4.8 (2.5)		0.292

*%TE* Percentage of Total Energy, *RNI* reference nutrient intake, *SD* Standard deviation, *IQR* interquartile Range, *g* gram *P values showing significant difference in nutrient intake for the two time points; ^a^ Department of Health (1991) and Scientific Advisory Committee on Nutrition (2012)

There were also differences in nutrient intakes by ethnicity. As shown in Table 3, South Asians reported significantly higher intakes of energy (*p* = 0.003) at both time points. Post-hoc analysis using the Turkey HSD test at baseline indicated that the mean score of energy intake of South Asian was significantly higher than that of African/Caribbean (*p* = 0.028). The mean energy intakes of the Other ethnicity was also significantly higher than that of African/Caribbean (*p* = 0.015). However, there was no significant difference between mean energy intakes of South Asians and Other ethnicities (*p* = 0.303). At follow-up, the post-hoc analysis showed a similar trend. The mean energy intakes of South Asians were significantly higher than that of African/Caribbean (*p* = 0.002). There was no significant difference between intakes of Other ethnicity and the mean energy intakes of South Asians (*p* = 0.571) or African/Caribbean (*p* = 0.589).

**Table 3:** Energy and macronutrient contribution to energy by ethnicity over time

	Baseline (***n*** = 100)				Follow-up (***n*** = 81)			
	South Asian (***n*** = 34)	African/Caribbean (***n*** = 60)	Others (***n*** = 6)	*P-value	South Asian (***n*** = 22)	African/ Caribbean (***n*** = 53)	Others (***n*** = 6)	*P-value
Energy kcal/day	1900.5	1615.8	1873.3	0.003	1969.8	1470.0	1641.5	0.003
%TE Carbs	48.6	49.0	43.9	0.625	48.3	55.4	53.0	0.141
%TE Sugars	12.1	15.1	17.4	0.119^a^	14.9	17.4	20.5	0.056 ^a^
%TE Protein	16.4	17.7	13.8	0.013	15.3	16.4	13.8	0.444 ^a^
%TE Fats	37.6	32.4	42.8	0.019	36.6	30.1	31.3	0.048
%TE Saturated fats	10.0	10.8	13.0	0.687	12.0	10.7	12.1	0.329
%TE Monounsaturated	11.1	11.3	13.4	0.385	10.8	10.4	11.3	0.768
%TE Polyunsaturated	7.2	4.9	7.0	< 0.001	8.1	4.3	5.6	< 0.001
Fibre intake g/day	16.0	15.0	20.6	0.256	17.2	15.9	20.3	0.285

*TE* Total energy; *P- value calculated using one-way ANOVA; ^a^ Kruskal Wallis used to calculate *P*-values

There was also reported differences in the intakes of %TE PUFA at both time points (*p* < 0.001). Post hoc analysis revealed that South Asians had significantly higher intakes than African/Caribbean at baseline (*p* < 0.001) and follow-up (*p* < 0.001). The %TE PUFA intakes of the Other ethnicity did not differ from South Asians (*p* = 0.597) or African/Caribbean (*p* = 0.256). Similarly, at follow-up there was no significant difference between Other ethnicity and South Asians (*p* = 0.067) or African/ Caribbean (*p* = 0.978).

Additionally, South Asians reported significantly higher intakes of %TE total fat at baseline (*p* = 0.019) and follow-up (*p* = 0.048). Post Hoc analysis revealed that South Asians %TE fats were significantly different from African/Caribbean at baseline (*p* = 0.038) and follow-up (*p* = 0.044). There was no significant difference between intakes of Other ethnicity and South Asians (*p* = 0.819) or African/Caribbean (*p* = 0.153) at baseline. At follow-up, it was a similar pattern, Other ethnicity intakes of %TE was not significantly different from South Asians (*p* = 0.302) or African/Caribbean (*p* = 0.987).

### Micronutrient intakes over time compared with RNIs

There were significant median decreases for only the following micronutrients: vitamin B6, vitamin B1, iron, folate and magnesium. In total, 39% (56% Female) and 35% (54% females) of participants reported taking micronutrient supplements during baseline and follow-up assessments, respectively. The most common supplements reported included multivitamins, vitamin C, vitamin D, iron and calcium. A sensitivity analyses revealed that for females at baseline, there were significant differences in intakes with and without supplementation in relation to vitamin D, vitamin A, vitamin E, thiamine, riboflavin, niacin, vitamin B6, vitamin B12, folate and vitamin C intakes (Additional file 3). For males, there were significant differences in the intakes of vitamin A, vitamin D, vitamin E, vitamin C and folate between intakes with supplementation and intakes without supplementation (Additional file 3). Micronutrient intakes with and without supplementation for females differed in vitamin A, vitamin D, vitamin B1, vitamin E vitamin B6, vitamin B12, vitamin C and manganese intakes at follow-up. However, there were no differences found for males (Additional file 4).

**Fig. 1:**
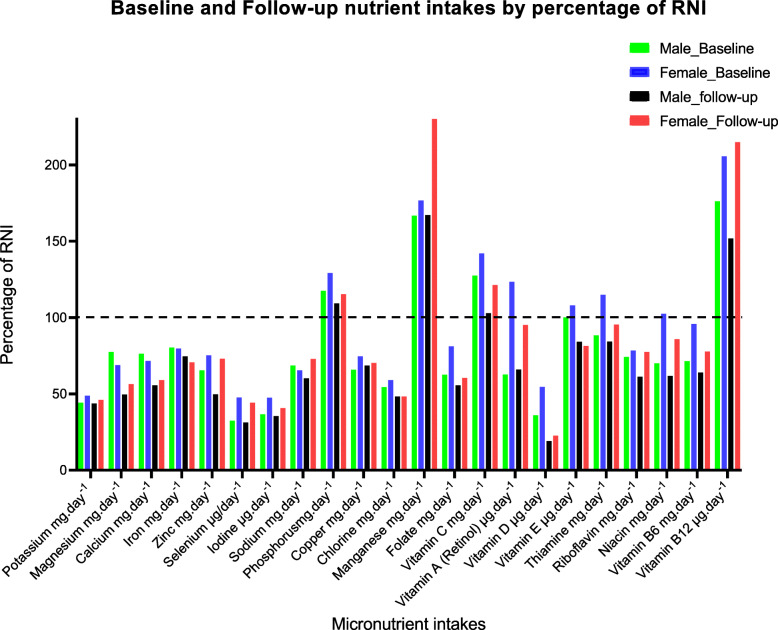
Percentage micronutrient intakes by UK RNI at baseline and follow-up

Compared to the UK RNI, total daily intake of most nutrients were below the recommendation. Expressed as a percentage of RNI (Fig. 1), daily intakes of vitamin B12, phosphorus and manganese for both sexes, and vitamin A, vitamin E, vitamin C, thiamine and niacin for females met the RNI. At follow-up, the pattern was similar for both sexes; except for phosphorus, manganese, vitamin C and vitamin B12, all other micronutrients were below the RNI.

### Cross-sectional associations

Baseline pairwise correlation found that SPPB was significantly associated with WC (*r* = − 0.466), MNA-SF (*r* = 0.391), BMI (*r* = − 0.224), HGS (*r* = 0.610), fibre (*r* = 0.265), vitamin B6 (*r* = 0.303) and vitamin D (*r* = 0.223). Nutritional status measured by MNA-SF was significantly associated with vitamin D (*r* = 0.237) and HGS (*r* = 0.198) (Additional file 5).

Using the results from the pairwise correlation, hierarchical multiple regression was conducted to determine the contribution of each independent variable to physical function performance and nutritional status. Table 4 shows the fully adjusted model for sociodemographic variables, nutritional status, WC, BMI and nutrient intakes as predictors of the SPPB and HGS.

**Table 4:** Hierarchical multiple regression predicting SPPB and HGS scores (*N* = 100)

	SPPB				HGS (kg)			
**Predictors**	**B (95% CI)**	**SE**	***p*-value**	*R*^*2*^*change*	**B (95% CI)**	**SE**	***p*-value**	*R*^*2*^*change*
**Sociodemographic variables**								
Male vs female	−0.05 (−1.11, 1.01)	0.53	0.930	0.216	−3.02 (−6.56, 0.52)	1.78	0.093	0.342
Age	−0.09 (− 0.15, − 0.03)	0.03	< 0.001		− 0.45 (− 0.65, − 0.25)	0.1	< 0.001	
Married vs not married	− 1.34 (−2.45, − 0.24)	0.55	0.020		−0.53 (− 1.30, 0.24)	0.39	0.177	
IMD	0.14 (−0.09, 0.37)	0.12	0.240		−1.41 (−2.66, −0.15)	0.63	0.028	
No education vs Education	−1.23 (−2.64, 0.18)	0.71	0.090		1.17 (−3.54, 5.88)	2.37	0.623	
Number of diseases	−0.26 (−0.64, 0.11)	0.19	0.170		−2.87 (−6.56, 0.82)	1.85	0.125	
**Nutritional status (by MNA-SF)**	0.39 (0.10, 0.67)	0.14	0.010	0.091	0.16 (−0.78, 1.10)	0.47	0.732	0.009
**WC**	−0.15 (− 0.22, − 0.09)	0.03	< 0.001	0.152	− 0.05 (− 0.52, 0.42)	0.24	0.834	0.010
**BMI**	0.12 (−0.02, 0.26)	0.07	0.090		−0.06 (− 0.28, 0.16)	0.11	0.579	
**Nutrient intakes**								
Fibre	0.08 (0.02, 0.15)	0.03	0.010	0.075	0.28 (0.07, 0.50)	0.11	0.011	0.061
Vitamin D	−0.04 (−0.12, 0.04)	0.04	0.340		−0.19 (− 0.45, 0.08)	0.13	0.164	
Vitamin B6	1.33 (0.35, 2.32)	0.49	0.010		1.33 (−1.95, 4.62)	1.65	0.421	
*Total R*^*2*^	0.534				0.423			

*N* 100, *WC* Waist Circumference, *IMD* Index of Multiple Deprivation, *BMI* Body Mass Index, *MNA-SF* Mini-Nutritional Assessment-Short Form

Given that only fibre, vitamin D and vitamin B6 were found to be correlated with SPPB and HGS, they were the only nutrients included in the multiple regression analyses. Higher SPPB scores were associated with being younger, married, a lower WC, and higher fibre and vitamin D intakes [*R*^*2*^ = 0.534, F (12, 87) =8.319, *p* < 0.01]. For HGS, increasing age was significantly associated with lower HGS scores. In addition, those living in the least deprived areas (determined by the IMD scores) and reporting higher fibre intakes had significantly higher HGS scores [*R*^*2*^ = .0423, F = (12, 87) =5.317, *p* < 0.01].

A hierarchical multiple regression was conducted to test the association of nutritional (MNA-SF) and physical function while controlling for all confounders (see Table 5). This was to determine the impact of nutrient intake and physical function (SPPB and HGS) as predictors of nutritional status. SPPB was the only predictor of nutritional status, with no other variables being significantly associated with nutritional status [*R*^*2*^ = .0278, F = (14, 85) = 2.334, *p* = 0.009]. After controlling for all confounders, higher SPPB scores were associated with better nutritional status.

**Table 5:** Hierarchical linear regression showing predictors of nutritional status (*N* = 100)

Predictors	B (95%CI)	SE	***P***-value	*R*^*2*^*change*
**Sociodemographic variables**				
Male vs female	0.17 (−0.61, 0.96)	0.39	0.664	0.107
Age	0.02 (−0.03, 0.07)	0.02	0.379	
Married vs not married	−0.34 (−1.16, 0.49)	0.41	0.420	
IMD	−0.14 (− 0.31, 0.03)	0.09	0.112	
No education vs Education	0.27 (−0.78, 1.33)	0.53	0.607	
Number of diseases	−0.24 (− 0.52, 0.03)	0.14	0.085	
WC	−0.02 (− 0.04, 0.07)	0.03	0.535	0.017
BMI	0.01 (−0.11, 0.10)	0.05	0.953	
**Physical function**				
SPPB	0.25 (0.08, 0.42)	0.09	0.004	0.096
HGS	−0.03 (−0.08, 0.03)	0.03	0.253	
**Nutrient intakes**				
Fibre	0.02 (−0.03, 0.06)	0.02	0.530	0.036
Vitamin D	−0.05 (− 0.11, 0.01)	0.03	0.092	
Vitamin B6	−0.15 (− 0.90, 0.59)	0.37	0.687	
*Total R*^*2*^	0.256			

*WC* Waist Circumference, *IMD* Index of Multiple Deprivation, *BMI* Body Mass Index, *HGS* Handgrip strength, *SPPB* Short Performance Physical Battery

### Longitudinal associations

There were significant changes in nutritional status measured using MNA-SF, and physical function using SPPB scores over time, (Z = -2.37, *p* = 0.018) and (Z = -4.01, *p* < 0.001) respectively. Per the MNA-SF cut-offs, 24% were malnourished/at-risk of malnutrition, and the remaining (76%) were normal at baseline. At follow-up, 37% were malnourished/at-risk of malnutrition, and remaining (63%) were normal. In summary, the proportion of people reporting normal status reduced, while those reporting malnourished/at-risk of malnutrition increased by 13% at follow-up. The changes in nutritional status were: 1) those who remained malnourished/at-risk of malnutrition at follow-up (12.3%); 2) those who changed to malnourished/at-risk of malnutrition at follow-up (24.7%); 3) those who changed to normal at follow-up (*n* = 8.6%); and 4) those who remained normal at follow-up (54.3%).

Using ‘remained normal at follow-up’ as the reference category in a multinomial regression (Table 6), the results indicate no significant predictors determining the likelihood of remaining malnourished/at-risk of malnutrition as compared to the reference group. However, participants with a higher SPPB score at baseline (OR = 0.61) were less likely to change to being malnourished/at-risk of malnutrition. Conversely, participants with a higher BMI (OR = 1.29) or WC (OR = 1.10) were more likely to change to being malnourished/at-risk of malnutrition compared to the reference group.

**Table 6:** Multinomial regression of factors predicting nutritional status membership at follow-up (*n* = 81)

	Remained At Risk/Malnourished			Changed to At-Risk/Malnourished			Changed to Normal		
	B	SE	OR (95% CI)	B	SE	OR (95% CI)	B	SE	OR (95% CI)
BMI	0.19	0.2	1.21 (0.81–1.80)	0.26	0.12	1.29 (1.01–1.65)*	0.29	0.31	1.34 (0.72–2.48)
HGS	−0.08	0.1	0.92 (0.75–1.13)	−0.16	0.07	0.85 (0.74–0.98)	−0.16	0.13	0.85 (0.66–1.11)*
WC	−0.12	0.1	0.89 (0.73–1.08)	0.09	0.06	1.10 (0.97–1.24)*	0.39	0.18	1.48 (1.04–2.10)
Age	0.06	0.09	1.07 (0.90–1.27)	0.12	0.06	1.13 (1.00–1.28)	0.36	0.14	1.43 (1.09–1.87)
IMD	0.21	0.46	1.23 (0.50–3.05)	0.40	0.19	1.49 (1.03–2.16)*	1	0.38	2.71 (1.30–5.68)*
SPPB	−0.26	0.32	0.77 (0.41–1.45)	−0.49	0.22	0.61 (0.40–0.94)*	− 0.85	0.45	0.43 (0.18–1.03)
Fibre	−0.28	0.15	0.75 (0.56–1.02)	−0.1	0.06	0.91 (0.81–1.02)	−0.07	0.11	0.93 (0.75–1.15)
Vitamin D	0.16	0.13	1.18 (0.92–1.51)	−0.05	0.1	0.95 (0.78–1.16)	0.12	0.18	1.13 (0.79–1.60)
Vitamin B6	1.17	1.53	3.23 (0.16–64.96)	0.05	0.78	1.05 (0.23–4.79)	0.11	1.44	1.12 (0.07–18.7)
Male	3.07	1.9	21.56 (0.52–892.87)	0.5	0.8	1.64 (0.34–7.90)	−1.53	1.86	0.22 (0.01–8.31)
Female	1			1			1		
Married	0.2	1.41	1.22 (0.08–19.22)	0.93	0.93	2.54 (0.41–15.67)	1.76	2.08	0.25 (0.10–3.43)
No married	1			1			1		
Educated	2.55	1.9	12.85 (0.31–527.7)	1.20	1.07	3.32 (0.41–26.83)	−16.41	0	0.743 (0.07–0.09)
No education	1			1			1		
Number diseases ≤2	−0.54	1.81	0.59 (0.02–20.21)	− 1.28	0.93	0.28 (0.05–1.71)	−2.18	1.9	0.25 (0.03–4.69)
Number diseases > 2	1			1			1		

Reference category: Remained Normal; *OR* Odds Ratio, *SE* Standard Error, *95% CI* Confidence Interval, *SPPB* Short Physical Performance Battery, *IMD* Index of Multiple Deprivation, *BMI* Body Mass Index, *R*^*2*^ = 0.63 (Nagelkerke) **P value < 0.01 *P value < 0.05

Regarding SPPB at follow-up, 35.8% remained stable, 47% had a lower score, and 17.2% improved their score. Computed into two groups, improved/stable and decline served as outcome variables in a logistic regression performed to determine the significant predictors of changes in SPPB at follow-up. The results indicate that with an increase in age (OR = 1.105, *p* = 0.005) or increase in the number of diseases reported (OR = 1.63, *P* = 0.033), participants were more likely to experience a decline in physical function than to improve or remain stable. All other variables showed no significant association with the changes in physical function (See details in Additional file 6).

## Discussion

This study provides unique and potentially useful evidence regarding the profile of nutrient intakes, nutritional status, and physical function, and their relationships over time, in a sample of ethnically diverse community-dwelling older adults. These findings are particularly relevant as they add to existing literature to broaden our understanding of the relationship between diet and components of healthy ageing within this population. Also, the findings are both timely and pertinent given the growing ethnic diversity in the UK and some parts of Europe, highlighting the need for culturally adapted interventions specifically around diet and physical function to support this group to age more healthfully [4, 41].

The intakes of energy and most nutrients remained unchanged, except decreases in the daily intakes of magnesium, folate, vitamin B1, vitamin B6 and iron. This finding concurs with a 10-year longitudinal study of 25 South Asian women (mean age of 54.2 years) in Glasgow [42]. This study used a 7-day weighed food dairy and found no significant difference in energy intake. However, the findings contrast sharply with the Melbourne Chinese Cohort Study, composed of 262 Chinese adults with a mean age > 50 years [43]. The authors found that intakes of energy, fats, fibre and protein increased significantly over 8 years among women, but not men. Among men, the only significant change was a decrease in carbohydrate intake. This study utilised FFQ, which has been found to overestimate nutrient intakes in older adults [27]. This, coupled with the failure of the authors to account for an increase in nutrient supplement intakes at follow-up, could partially account for the differences between these studies. Additionally, the relatively shorter duration of follow-up in the present study as compared to the Melbourne study could also contribute. Furthermore, as previously reported in this sample, eating patterns were linked to cultural and religious purposes rather than health reasons, which could account for the stability of energy and macronutrient intakes over time [44].

In the context of the broader literature, the energy intakes observed over time in this study were comparable with those of the energy intakes of predominately white older adults in the UK aged 65+ years as reported in the National Diet and Nutrition Survey (NDNS) [45]. Also, the energy intakes of males in the present study (1853.8 kcal/day baseline and 1822 kcal/day follow-up) were comparable to energy intakes of males in the Newcastle 85+ cohort (1848 kcal/day), but those of females (1358.5 kcal/day baseline and 1243.5 kcal/day follow-up) were less than the female energy intake values reported in the Newcastle 85+ cohort (1471 kcal/day) [46]. Additionally, the %TE from carbohydrates, protein, MUFA, and PUFA in the present study were higher, while the %TE from saturated fat was lower than the Newcastle 85+ cohort [46]. It has been observed that the traditional foods of ethnic minorities, with the exception of South Asian diets, are relatively higher in %TE from carbohydrate and lower in %TE from fats (including saturated fats), as compared to western diets [47, 48]. Hence, this difference in food composition between these ethnic groups and the disparity in age between the present sample and the Newcastle 85+ cohort might be accounting for the differences in energy and macronutrient intakes observed. The relatively high total energy and %TE from fats within the South Asian diets were also confirmed in this study, with participants identifying as South Asian having significantly higher intakes of total energy and %TE from fats as compared to those identifying as being African-Caribbean.

Another key finding from the present study is the inadequate amounts of micronutrients consumed. The intakes of these nutrients below the RNI were consistent at follow-up, suggesting a potential micronutrient deficiency in this sample. Given the importance of micronutrients for maintenance of good health, physical function and better quality of life, the low intakes are of concern, particularly when one considers the relatively high prevalence of non-communicable disease within this population [10]. The inadequate intake of various nutrients among older ethnic minorities in the present study is consistent with previous studies in the UK and elsewhere [11, 49]. However, in contrast to the present findings, the results from Years 7 and 8 (combined) of the UK NDNS Rolling Programme (a sample comprised of predominately white older adults), found that intakes of most micronutrients were at the RNI or above the RNI [45]. Variations in the level of deprivation between these two samples could be one factor accounting for these differences. It has been reported elsewhere that poverty and other forms of deprivation lead to poorer diets, and subsequently, a higher prevalence of malnutrition, in both community and hospital settings [50, 51]. Recently, the English Longitudinal Study of Ageing reported that being non-white (OR: 3.8; 95% CI 2.39–6.05) and obese (OR: 1.32; 96% CI 1.09–1.58) were associated with a higher vitamin D deficiency [52]. These findings further highlight the inequalities in nutrient intakes and the need for increasingly culturally tailored community-focussed interventions to promote adequate nutritional intake and the general health of this population.

Given the critical role of supplementation and the increased tendency of poorer nutrition in later life, nutrient supplementation is beneficial to healthy ageing [53, 54]. For instance, a meta-analysis of randomised controlled trials concluded that supplementation of 17.5–25 μg of vitamin D daily reduces the risk of falls by 19% in older adults [54]. The vital role of nutrient supplementation observed in this study adds to the literature; supplementation significantly contributed to meeting the RNI for nutrients such as vitamin A, vitamin D, vitamin B6 and vitamin B12, thiamine, niacin and vitamin E. However, the consumption of supplementation was relatively low over time, and lower for males as compared to females. Further studies explicitly examining prolonged supplementation within this population is needed to confirm our findings. However, given the benefits of supplementation observed, this population might benefit considerably from micronutrient supplementation.

Nutritional status and physical function declined significantly over the study period. The findings showed an increase of 13% in the number of people classified as malnourished or at-risk of malnutrition over the study period. Likewise, over time, almost half (47%) experienced at least one unit decrease in SPPB scores. These declines over a relatively short period are disturbing, which highlights the need for timely community nutritional and physical function assessment and appropriate strategies to address declines. The prevalence of malnutrition or at risk of malnutrition at both time points within this present study was more than double the estimated prevalence of malnutrition in the UK. It is estimated that more than 3 million older adults suffer from malnutrition, which accounts for more than 10% of the population aged 65 years and older [55, 56]. However, as seasonality is known to impact on eating behaviours and physical function, the season in which data were collected could have affected participants’ nutrient intake and subsequent nutritional status, leading to the high proportion of malnourished/at risk of malnutrition observed among this sample [44]. Despite this, the prevalence of malnutrition or at risk of malnutrition in studies outside the UK reported considerably higher percentages than the current study [57, 58]. For example, a study of 360 older adults aged 60 years and over in India found that 70% of the sample were malnourished or at risk of malnutrition [57]. In comparing the prevalence of malnutrition of the present study with existing literature, it is important to note the use of different tools in assessing nutritional status. The MNA-SF used in the present study has been shown to have a specificity of 100%, sensitivity of 97.9%, and diagnostic accuracy of 98.7% in undernutrition prediction as compared to the MNA-long version [32]. Further validation of the MNA-SF against the MNA-SF in a recent systematic review and meta-analysis found that the MNA-SF (cut-off point ≤11) had a sensitivity of 0.95 (0.75–0.99) and specificity of 0.95 (0.85–0.99) in detecting community-dwelling older adults at risk of malnutrition [59]. Additionally, a study of 155 older adults with a mean age of 78 years exploring the relationship between the MNA-SF and other comprehensive nutritional assessment measures, found that the MNA-SF had a stronger correlation with anthropometric and biological markers (*p* < 0.01) [60]. Considering the accuracy, the non-invasiveness and cost effectiveness of the MNA-SF, it is often regarded as the preferred tool to be used in the identification of malnutrition in the community.

The positive relationship between physical function and nutritional status in the present study confirms the findings of previous studies [61, 62]. A study of 457 randomly selected older Bangladeshis living in rural communities found that poorer nutritional status, increased age, existence of co-morbidities, and being female were associated with functional limitations [58]. One possible explanation for this relationship could be the difficulty in shopping and preparing food, especially their traditional meals, due to physical limitations, which could eventually lead to inadequate dietary intake and malnutrition, which further exacerbates their physical function limitations. Additionally, frailty as a related concept to physical function could have influenced the observed relationship between nutritional status and physical function in the present study. A recent systematic review and meta-analysis found a strong positive association and considerable overlap, of 47%, between frailty and malnutrition of hospitalised older adults [63]. As earlier explained, physical frailty affecting mobility could have adverse consequences on how older adults shop for food and prepare healthy meals, which could further exacerbate their nutritional status, and in a cyclical manner, worsen their frailty status. Physical function measured using SPPB also serves as an indicator of risk of frailty, and proposed cut-offs of SPPB scores are often used to assess frailty status. In the present study, using the summary cut-offs of SPPB scores proposed by the European Medicines Agency, 22% of the sample were considered frail, 14% pre-fail and the majority (64%) considered non-frail at baseline [64]. In addition, almost half (43.3%) of the frail and pre-frail older adults, as indicated by SPPB score, were found to be malnourished or at risk of malnutrition. Among the non-frail older adults, 78.4% were found to have normal nutritional status. These findings substantiate the correlation and overlap between frailty and malnutrition, as reported in previous studies [63, 65].

There was no significant relationship between sex and nutritional status in this present study. The role of women in society and their relative financial dependency partly accounts for the differences in nutritional status as reported by previous studies [57, 66]. However, within this study, there was no difference in education and deprivation (IMD scores) between sexes, suggesting that females may have been more comparable to males with regards to resources and access to healthier foods, and hence could afford to cook and eat diets similar to their male counterparts. Also, it could be assumed that by virtue of western economic culture and the availability of supermarkets in the Birmingham area, females had fairly equal access to food as compared to traditional customs of some cultures where men eat first, and older women tend to give their share of their food to other family members, especially their grandchildren [62].

The strengths of this study include the use of the multiple-pass 24-h recall approach on four-non-consecutive days (including a weekend day) compared to the use of a single 24-h recall by previous studies which fails to account for day-to-day variation, or the FFQ which has been found to overestimate energy and nutrient intakes in older adults [11, 27, 67]. However, it is important to acknowledge the high possibility of under-reporting and recall bias in this study. Under-reporting contributes to a significant amount of error in dietary reporting and is even higher in overweight and obese populations, such as those participating in the present study [68]. Additionally, the number of days used in assessing diet in this present study was insufficient to capture some nutrients. Studies have reported a range of 3 to 9 days of 24-h recalls to capture a true representation of energy and macronutrients, and a much wider range of 4 to 160 days of 24-h recalls to capture a true representation of micronutrient intakes [69].

Furthermore, given the influence of seasonality and increasing age on dietary intake and nutritional status, the 8 months’ follow-up period within this present study may have been relatively short to assess the changes in these variables accurately. Hence, one must exercise caution in drawing any firm conclusions from these findings, as they can only provide an insight into the potential changes in dietary trends and nutritional status over time within this population. Future studies with more extended follow-up periods, which control for seasonality, are required to investigate the dietary trends and nutritional status within this population. Lastly, even though the maximum variation technique was used during sampling to ensure a more diverse sample, it was challenging to recruit South Asian females, especially females self-identifying as Bangladeshi. Thus, the findings are not generalizable to all ethnic minorities living in the UK.

### Implications for research and practice

Given the declines in nutritional status and physical function observed within a relatively short period, strategies for implementing a community-led programme around early nutritional and physical function assessments at community and faith/religious centres could help to identify and treat malnutrition and physical function limitations before further deterioration. The co-creation of such programmes with health care professionals and community/faith leaders may be effective in increasing accessibility and acceptability of these assessments, and also ensure that consistent health messages are delivered across communities. Interventions that are co-created and run at faith centres have been shown to increase access and improve health outcomes [70, 71]. These benefits can serve as a strong incentive for community-dwelling older adults to visit these meeting places to improve their social networks, which is equally essential for a healthier ageing trajectory.

As previously reported in this same sample, fear of gaining weight and engagement in unhealthy practices to lose weight were observed [44]. The practice of fasting, skipping meals, or drastically reducing portion sizes without proper adherence to diet quality are common practices, which may have contributed to the observed low nutrient intakes in this study. While future studies are recommended to explore this in detail, health professionals could change their advice and avoid generic messages such as “you are overweight/obese, so you need to lose weight,” and instead deliver more individually tailored messages to achieve weight loss using healthier practices that do not negatively impact nutritional quality of one’s diet.

Lastly, given the importance of supplementation and the low supplement intakes observed in this study, this population would benefit from a free supplementation programme [72, 73]. To increase compliance, such a programme could be integrated into already existing community or faith group activities. Additionally, health professionals, specifically nutritionists and dieticians attending to older ethnic minorities, could include a mandatory dietary assessment and based on the results, prescribe free supplements if needed, in line with safer intake thresholds, to enhance acceptability and intake to improve nutrient status among this population. This practice could be complemented with appropriately tailored dietary education to ensure that these supplements do not replace a well-balanced diet.

## Conclusion

The present findings indicate that community-dwelling, ethnically diverse older adults consume most micronutrients below the UK RNI over time. In a relatively short period of follow-up, there were significant declines in nutrient intakes (folate, vitamin B1, vitamin B6, magnesium and iron), nutritional status and physical function among this population. These declines, and the observed associations between nutritional status and physical function over time highlight the need for enhanced community programmes targeting early screening of nutritional status and physical function to ensure most older adults are assessed and treated accordingly. Additionally, the inclusion of dietary assessments, provision of free dietary supplements and culturally tailored dietary education by health professionals could improve nutritional intake among this population. Future studies with a longer follow-up period and larger sample size are recommended to confirm the findings of this study.

## Supplementary information

**Additional file 1.** Bespoke socio-demographic questionnaire.

**Additional file 2 **Differences between participants that remained in the study (*n* = 81) and participants that dropped out (*n* = 19).

**Additional file 3 **Differences in micronutrient intakes with and without supplementation at baseline (*n* = 100).

**Additional file 4 **Differences in micronutrient intakes with and without supplementation at follow-up (*n* = 81).

**Additional file 5 **Pairwise correlations at baseline (*n* = 81).

**Additional file 6.** Predictors of changes in physical function (SPPB) over time.

## Data Availability

The datasets generated and analysed during the current study are part of a larger PANINI network data set containing unpublished data, hence are not currently available. The data will be made available after completion of the Innovative Training Network (ITN) project.

## Abbreviations

WC: Waist Circumference
CI: Confidence interval
SD: Standard Deviation
IMD: Index of Multiple Deprivation
MNA-SF: Mini-Nutritional Assessment-Short Form
SPPB: Short Physical Performance Battery
HGS: Handgrip strength
BMI: Body Mass Index
