# Supplementary material for: The association between nutrient intake, nutritional status and physical function of community-dwelling ethnically diverse older adults
Source: BMC Nutr. 2020 Aug 25;6:36. doi: 10.1186/s40795-020-00363-6 (PMC7447572; doi:10.1186/s40795-020-00363-6)
Supplement: Supplementary file 1 — Additional file 1. Bespoke socio-demographic questionnaire. [file 40795_2020_363_MOESM1_ESM.docx]

### **Additional file 1 . Bespoke socio-demographic questionnaire**

Participant ID: _________________ Date: _____________________

*Interviewer: Please explain to the participant that the following questions are to gather general information about her age, origin, education, household, and health. Please tick only one box for each question.*

| **1. GENERAL INFORMATION**  **Age:** ____________________DOB:_________ |  | |
| --- | --- | --- |
| **Country of birth:** _______________________ |  | |
| **Ethnicity**  *Mixed/ Multiple ethnic backgrounds*  White and Black Caribbean  White and Black African  White and Black Asian  Other __________________________  *Asian*  Indian  Pakistani  Bangladeshi  Other __________________________  *Black/ African/ Caribbean*  African  Caribbean  Other__________________________  Any other ______________________ |  | |
| **Marital status**  Single  Married  Separated  Divorced  Widowed |  | |
| **Number of children** _________________________ |  | |
| **How long have you been living in the UK?** _____________ |  | |
| **Education**  No qualifications  Primary school  Secondary school  College  University/Higher education |  | |
| **Faith/Religion**  Buddhist  Christian  Hindu  Jew  Muslim  Sikh  No religion  Other (please state)  **2. HOUSEHOLD**  Postcode _________________________ |  | |
| How long have you been living at this address?  How many people are currently living in your household, including yourself? ____________ | | |
|  | |  |
| **Do you work for pay outside the home?** ……………………  If yes, check the box that best corresponds to you  Working full time ……………………………………………………  Working part time …………………………………………………..  Have you ever worked in the UK?  If yes, are you retired? | Yes  No      Yes  No  Yes  No | |
| **3. HEALTH**  **How would you describe your health compared to other persons of your age?**  Excellent …………………………………………………………….  Good …………………………………………………………………  Fair …………………………………………………………………...  Poor………………………………………………………………….. |  | |
| **Have you been diagnosed with any of these diseases in the past**? …  Hypertension ……………………………………………………...  Diabetes ……………………………………………………………  Heart disease and stroke.………………………………………..  Hyperlipidaemia ……………………………………………………  Renal disease …………………………………………………….  Osteoporosis ………………………………………………………  Arthritis …………………………………………………………….  Cancer ……………………………………………………………..  Other: ____________________________________________ | Yes  No | |
| **Do you take any medication?** Yes  No  **Description:** ______________________________________________________________ | | |
| **Do you currently smoke?**  Frequency_______cigarettes/week  Did you use to smoke? For how long (years)? _________ | Yes  No  Yes  No | |

| **Do you consume drinks containing alcohol?**  Frequency __________ /week Type________ | Yes  No |
| --- | --- |
