# Supplementary material for: The association between nutrient intake, nutritional status and physical function of community-dwelling ethnically diverse older adults
Source: BMC Nutr. 2020 Aug 25;6:36. doi: 10.1186/s40795-020-00363-6 (PMC7447572; doi:10.1186/s40795-020-00363-6)
Supplement: Supplementary file 2 — Additional file 2 Differences between participants that remained in the study (n = 81) and participants that dropped out (n = 19). [file 40795_2020_363_MOESM2_ESM.docx]

**Additional file 2: Differences between participants that remained in the study (n=81) and participants that dropped out (n=19)**

| **Variables** | | **Completed study (n= 81)** | **Lost to follow-up (n= 19)** | **P-value** |
| --- | --- | --- | --- | --- |
| Age (median) | | 70 | 71 | 0.878 |
| Sex (N) | Males | 50 | 9 | 0.252 |
|  | Females | 31 | 10 |  |
| Marital status (N) | Married | 55 | 11 | 0.410 |
|  | Not married | 26 | 8 |  |
| Education (N) | Educated | 70 | 14 | 0.175 |
|  | Not educated | 11 | 5 |  |
| IMD (median) | | 2.7 | 3.1 | 0.121 |
| Number of diseases (median) | | 2.0 | 2.1 | 0.141 |
| BMI (mean) | | 29.5 | 29.7 | 0.882 |
| WC (median) | | 99.2 | 106.1 | 0.036 |
| MNA-SF (median) | | 13.1 | 13 | 0.404 |
| SPPB (Median) | | 11.1 | 8 | 0.007 |
| HGS (kg) (mean) | | 27.9 | 22.5 | 0.027 |
| Energy kcal (median) | | 1733 | 1878 | 0.186 |
