# Supplementary material for: The association between nutrient intake, nutritional status and physical function of community-dwelling ethnically diverse older adults
Source: BMC Nutr. 2020 Aug 25;6:36. doi: 10.1186/s40795-020-00363-6 (PMC7447572; doi:10.1186/s40795-020-00363-6)
Supplement: Supplementary file 3 — Additional file 3 Differences in micronutrient intakes with and without supplementation at baseline (n = 100). [file 40795_2020_363_MOESM3_ESM.docx]

**Additional file 3: Differences in micronutrient intakes with and without supplementation at baseline (n=100)**

|  | **Male (% of RNI)** | | | **Female (% of RNI)** | | |
| --- | --- | --- | --- | --- | --- | --- |
| **Micronutrients** | **Supplements** | **Without supplements** | **p-value**** | **Supplements** | **Without supplements** | **p-value**** |
| Potassium mg/day | 43.92 | 43.92 | 1.000 | 48.67 | 48.65 | 0.323 ^a^ |
| Magnesium mg/day | 77.24 | 50.91 | 0.109 | 68.65 | 67.71 | 0.180 |
| Calcium mg/day | 76.07 | 53.48 | 0.109 | 71.37 | 70.12 | 0.144 |
| Iron mg/day | 80.09 | 78.24 | 0.119 | 79.48 | 74.82 | 0.068 |
| Zinc mg/day | 65.33 | 61.48 | 0.059 | 75.05 | 72.26 | 0.655 |
| Selenium µg/day | 32.26 | 32.26 | 1.000 | 47.43 | 46.85 | 0.317 |
| Iodine µg/day | 36.44 | 33.44 | 0.980 | 47.27 | 45.78 | 0.317 |
| Sodium mg/day | 68.31 | 68.31 | 1.000 ^a^ | 65.31 | 65.31 | 1.00 |
| Phosphorus mg/day | 117.36 | 117.34 | 0.321 ^a^ | 128.93 | 128.83 | 0.323 ^a^ |
| Copper mg/day | 65.68 | 65.68 | 1.000 | 74.39 | 73.37 | 0.317 |
| Chlorine mg/day | 54.24 | 54.24 | 0.625 ^a^ | 58.82 | 58.8 | 0.317 |
| Manganese mg/day | 166.59 | 166.59 | 0.998 | 176.48 | 174.04 | 0.317 |
| Folate mg/day | 62.38 | 58.77 | 0.043 | 81.01 | 68.05 | 0.012 |
| Vitamin C mg/day | 127.36 | 122 | 0.043 | 141.83 | 120.65 | 0.012 |
| Vitamin A (Retinol) µg/day | 62.49 | 57.14 | 0.043 | 123.2 | 104.92 | 0.008 |
| Vitamin D µg/day | 35.81 | 21.86 | 0.008 | 54.34 | 26.1 | 0.001 |
| Vitamin E µg/day | 99.83 | 83.83 | 0.018 | 107.71 | 68.83 | 0.003 |
| Thiamine mg/day | 88.14 | 93.43 | 0.066 | 114.63 | 96.34 | 0.011 |
| Riboflavin mg/day | 74 | 63 | 0.066 | 78.12 | 73.19 | 0.012 |
| Niacin mg/day | 69.74 | 65.9 | 0.068 | 102.3 | 86.77 | 0.012 |
| Vitamin B6 mg/day | 71.19 | 66.1 | 0.068 | 95.53 | 78.46 | 0.011 |
| Vitamin B12 µg/day | 175.93 | 173.56 | 0.066 | 205.37 | 176.75 | 0.011 |

**p-values were calculated using absolute values controlled for energy intake and not computed percentages **^a^** Normally distributed dated, p-values were calculated using paired t-test analysis.
