# Supplementary material for: The association between nutrient intake, nutritional status and physical function of community-dwelling ethnically diverse older adults
Source: BMC Nutr. 2020 Aug 25;6:36. doi: 10.1186/s40795-020-00363-6 (PMC7447572; doi:10.1186/s40795-020-00363-6)
Supplement: Supplementary file 4 — Additional file 4 Differences in micronutrient intakes with and without supplementation at follow-up (n = 81). [file 40795_2020_363_MOESM4_ESM.docx]

**Additional file 4: Differences in micronutrient intakes with and without supplementation at follow-up (n=81)**

|  | **Male (% of RNI)** | | | **Female (% of RNI)** | | |
| --- | --- | --- | --- | --- | --- | --- |
| **Micronutrients** | **Supplements** | **Without supplements** | **p-value**** | **Supplements** | **Without supplements** | **p-value**** |
| Potassium mg/day | 43.47 | 43.32 | 0.123 ^a^ | 45.89 | 45.58 | 0.132 ^a^ |
| Magnesium mg/day | 49.37 | 49.68 | 0.229 ^a^ | 56.18 | 56.21 | 0.723 ^a^ |
| Calcium mg/day | 55.38 | 55.01 | 0.237 ^a^ | 58.73 | 57.2 | 0.322 ^a^ |
| Iron mg/day | 74.3 | 72.45 | 0.455 | 70.46 | 69.13 | 0.212 |
| Zinc mg/day | 49.53 | 49.56 | 0.577 | 72.8 | 69.15 | 0.434 |
| Selenium µg/day | 30.98 | 30.86 | 0.987 | 43.98 | 43.17 | 0.280 |
| Iodine µg/day | 35.23 | 35.26 | 0.090 | 40.6 | 38.76 | 0.242 |
| Sodium mg/day | 59.95 | 61.05 | 0.462 | 72.65 | 72.08 | 0.932 |
| Phosphorus mg/day | 109.12 | 108.29 | 0.228 ^a^ | 115.02 | 113.94 | 0.128 ^a^ |
| Copper mg/day | 68.37 | 68.11 | 0.352 | 70.1 | 68.18 | 0.490 |
| Chlorine mg/day | 48.09 | 47.89 | 0.588 | 48.09 | 47.89 | 0.571 |
| Manganese mg/day | 166.91 | 166.56 | 0.521 | 311.34 | 307.71 | 0.017 ^a^ |
| Folate mg/day | 55.32 | 54.79 | 0.541 ^a^ | 60.19 | 57.36 | 0.122 ^a^ |
| Vitamin C mg/day | 102.71 | 101.39 | 0.061 | 121.23 | 114.03 | 0.021 |
| Vitamin A (Retinol) µg/day | 65.75 | 63.05 | 0.256 | 94.92 | 82.05 | 0.027 |
| Vitamin D µg/day | 18.86 | 17.56 | 0.641 | 22.48 | 17.6 | 0.012 |
| Vitamin E µg/day | 83.96 | 83.63 | 0.795 | 81.18 | 78.68 | 0.025 ^a^ |
| Thiamine mg/day | 84.13 | 78.46 | 0.914 | 95.19 | 91.63 | 0.005 |
| Riboflavin mg/day | 61.07 | 59.28 | 0.169 | 77.27 | 72.66 | 0.089 |
| Niacin mg/day | 61.59 | 53.79 | 0.102 | 85.63 | 80.95 | 0.174 ^a^ |
| Vitamin B6 mg/day | 63.7 | 61.71 | 0.122 | 77.6 | 70.56 | 0.016 ^a^ |
| Vitamin B12 µg/day | 151.7 | 147.13 | 0.195 | 214.67 | 111.74 | 0.031 |

**p-values were calculated using absolute values controlled for energy intake and not computed percentages **^a^** Normally distributed dated, p-values were calculated using paired t-test analysis.
