# Supplementary material for: The association between nutrient intake, nutritional status and physical function of community-dwelling ethnically diverse older adults
Source: BMC Nutr. 2020 Aug 25;6:36. doi: 10.1186/s40795-020-00363-6 (PMC7447572; doi:10.1186/s40795-020-00363-6)
Supplement: Supplementary file 5 — Additional file 5 Pairwise correlations at baseline (n = 81). [file 40795_2020_363_MOESM5_ESM.docx]

**Additional file 5: Pairwise correlations at baseline (n=81)**

|  |  | **1** | **2** | **3** | **4** | **5** | **6** | **7** | **8** | **9** | **10** | **11** | **12** | **13** | **14** | **15** | **16** | **17** | **18** | **19** | **20** | **21** | **22** |
| --- | --- | --- | --- | --- | --- | --- | --- | --- | --- | --- | --- | --- | --- | --- | --- | --- | --- | --- | --- | --- | --- | --- | --- |
| **1** | BM1 | 1 |  |  |  |  |  |  |  |  |  |  |  |  |  |  |  |  |  |  |  |  |  |
| **2** | Hand grip | -0.131 | 1 |  |  |  |  |  |  |  |  |  |  |  |  |  |  |  |  |  |  |  |  |
| **3** | SPPB | -.224^*^ | .610^**^ | 1 |  |  |  |  |  |  |  |  |  |  |  |  |  |  |  |  |  |  |  |
| **4** | WC | .677^**^ | -0.169 | -.466^**^ | 1 |  |  |  |  |  |  |  |  |  |  |  |  |  |  |  |  |  |  |
| **5** | MNA-SF | -0.074 | .198^*^ | .391^**^ | -0.182 | 1 |  |  |  |  |  |  |  |  |  |  |  |  |  |  |  |  |  |
| **6** | Energy | 0.057 | 0.094 | 0.080 | 0.058 | 0.034 | 1 |  |  |  |  |  |  |  |  |  |  |  |  |  |  |  |  |
| **7** | Protein | 0.009 | -0.032 | 0.080 | 0.007 | -0.100 | .722^**^ | 1 |  |  |  |  |  |  |  |  |  |  |  |  |  |  |  |
| **8** | Fibre | -0.069 | .279^**^ | .265^**^ | -0.057 | 0.188 | .237^*^ | 0.065 | 1 |  |  |  |  |  |  |  |  |  |  |  |  |  |  |
| **9** | Potassium | -0.162 | 0.101 | 0.179 | -0.134 | -0.012 | -.397^**^ | -0.165 | .386^**^ | 1 |  |  |  |  |  |  |  |  |  |  |  |  |  |
| **10** | Calcium | 0.076 | -0.011 | -0.009 | 0.085 | -0.176 | -0.127 | -0.033 | 0.061 | .510^**^ | 1 |  |  |  |  |  |  |  |  |  |  |  |  |
| **11** | Magnesium | -0.149 | 0.106 | 0.139 | -0.070 | 0.100 | -.357^**^ | -.253^*^ | .445^**^ | .751^**^ | .478^**^ | 1 |  |  |  |  |  |  |  |  |  |  |  |
| **12** | Iron | -0.153 | -0.083 | -0.001 | 0.048 | -0.135 | -0.158 | -0.076 | .321^**^ | .481^**^ | .316^**^ | .610^**^ | 1 |  |  |  |  |  |  |  |  |  |  |
| **13** | Zinc | 0.000 | -0.187 | -0.122 | 0.092 | -0.154 | -0.104 | .254^*^ | -0.079 | .239^*^ | .200^*^ | .330^**^ | .450^**^ | 1 |  |  |  |  |  |  |  |  |  |
| **14** | Vitamin A | 0.084 | -0.038 | 0.047 | -0.005 | 0.059 | 0.008 | 0.112 | 0.037 | 0.008 | 0.054 | 0.119 | 0.091 | 0.114 | 1 |  |  |  |  |  |  |  |  |
| **15** | Vitamin D | -0.027 | 0.124 | .223^*^ | -0.084 | .237^*^ | -0.096 | 0.073 | 0.060 | 0.174 | 0.074 | 0.001 | -.211^*^ | -0.142 | 0.065 | 1 |  |  |  |  |  |  |  |
| **16** | Riboflavin | 0.067 | 0.021 | 0.114 | 0.101 | -0.050 | -0.128 | 0.086 | -0.005 | .435^**^ | .608^**^ | .354^**^ | .222^*^ | .266^**^ | .286^**^ | .202^*^ | 1 |  |  |  |  |  |  |
| **17** | Niacin | -0.069 | 0.065 | 0.177 | -0.069 | 0.029 | -0.128 | .279^**^ | -0.074 | .273^**^ | -0.028 | 0.081 | 0.009 | 0.124 | -0.057 | .243^*^ | .413^**^ | 1 |  |  |  |  |  |
| **18** | VitaminB6 | -0.114 | 0.164 | .303^**^ | -0.068 | 0.170 | -.284^**^ | -0.038 | 0.154 | .619^**^ | .227^*^ | .497^**^ | .268^**^ | 0.168 | -0.051 | .212^*^ | .576^**^ | .690^**^ | 1 |  |  |  |  |
| **19** | VitaminB12 | -0.082 | -0.082 | 0.092 | -0.090 | -0.039 | -0.045 | .310^**^ | -0.139 | 0.171 | 0.162 | -0.111 | -0.045 | 0.157 | .249^*^ | .423^**^ | .337^**^ | .223^*^ | 0.080 | 1 |  |  |  |
| **20** | Sodium | 0.195 | 0.012 | -0.161 | 0.040 | -0.195 | -0.042 | -0.053 | 0.017 | 0.178 | .230^*^ | 0.135 | 0.127 | -0.022 | 0.044 | -0.085 | 0.112 | 0.073 | 0.036 | -0.033 | 1 |  |  |
| **21** | Vitamin C | -0.148 | 0.177 | 0.153 | -0.133 | 0.047 | -.253^*^ | -.242^*^ | .383^**^ | .511^**^ | 0.189 | .378^**^ | .330^**^ | 0.003 | 0.021 | 0.009 | .268^**^ | 0.182 | .485^**^ | 0.026 | 0.189 | 1 |  |
| **22** | Folate | -0.124 | 0.152 | 0.188 | -0.034 | 0.031 | -.230^*^ | -0.159 | .417^**^ | .492^**^ | .336^**^ | .608^**^ | .524^**^ | 0.105 | 0.117 | 0.084 | .473^**^ | .211^*^ | .597^**^ | 0.032 | 0.017 | .498^**^ | 1 |
