# Supplementary material for: The association between nutrient intake, nutritional status and physical function of community-dwelling ethnically diverse older adults
Source: BMC Nutr. 2020 Aug 25;6:36. doi: 10.1186/s40795-020-00363-6 (PMC7447572; doi:10.1186/s40795-020-00363-6)
Supplement: Supplementary file 6 — Additional file 6. Predictors of changes in physical function (SPPB) over time. [file 40795_2020_363_MOESM6_ESM.docx]

**Additional file 6: Predictors of changes in physical function (SPPB) over time**

|  | **OR** | **95% CI** | **SE (B)** | **P-value** |
| --- | --- | --- | --- | --- |
| Constant | 0 |  | 5.26 | 0.007 |
| Male vs Female | 1.54 | 0.46-5.17 | 0.62 | 0.486 |
| Age | 1.10 | 1.03-1.18 | 0.04 | 0.005 |
| Married vs Not married | 0.91 | 0.25-3.34 | 0.66 | 0.885 |
| IMD | 1.02 | 0.78-1.33 | 0.14 | 0.908 |
| Educated vs Not educated | 1.98 | 0.35 -1.27 | 0.89 | 0.443 |
| Number of diseases | 1.63 | 1.04-2.55 | 0.23 | 0.033 |
| WC | 1.08 | 0.99-1.17 | 0.04 | 0.072 |
| BMI | 0.84 | 0.70-1.02 | 0.10 | 0.081 |
| MNA-SF | 1.21 | 0.84-1.74 | 0.19 | 0.302 |
| Fibre | 1.03 | 0.96-1.11 | 0.04 | 0.412 |
| Vitamin D | 1.05 | 0.95-1.15 | 0.05 | 0.373 |
| Vitamin B6 | 0.99 | 0.35-2.86 | 0.54 | 0.994 |
